# The Italian Version of the Adult Vaccine Hesitancy Scale (aVHS) for the Working-Age Population: Cross-Cultural Adaptation, Reliability, and Validity

**DOI:** 10.3390/vaccines10020224

**Published:** 2022-01-31

**Authors:** Caterina Ledda, Claudio Costantino, Giuseppe Liberti, Venerando Rapisarda

**Affiliations:** 1 Occupational Medicine, Department of Clinical and Experimental Medicine, University of Catania, 95100 Catania, Italy; vrapisarda@unict.it; 2 Department of Health Promotion, Mother and Child Care, Internal Medicine and Medical Specialties “G. D’Alessandro”, University of Palermo, 90100 Palermo, Italy; claudio.costantino01@unipa.it; 3 Commissioner Office in Acta for the COVID-19 Emergency, Provincial Health Authority of Catania, 95100 Catania, Italy; commissario.covid@aspct.it

**Keywords:** vaccination, workplace, SARS-CoV-2, vaccine hesitancy, Italy, worker, COVID-19, vaccine preventable disease

## Abstract

The adult Vaccine Hesitancy Scale (aVHS) is valid and reliable for evaluating attitudes toward vaccine preventable diseases (VPDs). The aim of the present study was to evaluate the cross-cultural adaptation, reliability, and validity of the Italian version of the aVHS. After cross-cultural adaptation of the aVHS, internal consistency (IC), intra-class correlation (ICC), and content validity (S-CVI) were evaluated through a survey on 160 workers. Results of the ICC were analyzed on questionnaires administered twice at a distance of two months and revealed a satisfactory reproducibility (0.87). The IC of the aVHS was assessed by the Cronbach alpha coefficient test, with a result of 0.94, demonstrating an excellent IC reliability. The S-CVI calculated for the total scale was 0.97. The aVHS is a valid and reliable tool for evaluating vaccine hesitancy toward adult vaccinations. We suggest the use of this scale in upcoming surveys on opinions and perceptions of adult vaccinations.

## 1. Introduction

Fighting the COVID-19 pandemic with a vaccine is essential for public and economic well-being. Nevertheless, realizing this depends on vaccine safety and efficacy and the strength of public trust in the vaccine. 

Vaccine hesitancy is not a new trend, dating as far back as the 19th century when Edward Jenner developed the smallpox vaccine in England [1]. 

Despite technological advances, vaccine hesitancy has grown into a progressively crucial concern in the 21st century, menacing existing herd immunity to highly predominant infections and slowing down improvements toward ongoing disease prevention measures [1,2]. According to the World Health Organization’s Strategic Advisory Group of Experts (WHO SAGE), vaccine hesitancy is now defined as “the delay in acceptance or refusal of vaccination despite the availability of vaccine services. Vaccine hesitancy is complex and context-specific, varying across time, place, and vaccines. It is influenced by factors such as complacency, convenience, and confidence” [3]. The outcomes of vaccine hesitation were in decline in pediatric vaccinations and the shift away from recommended vaccinations in some groups of workers at high risk of contagion and spread, such as healthcare personnel (HCP) [4,5,6,7,8,9,10,11,12]. 

In just under a year since the start of the SARS-CoV-2 pandemic, vaccines have been safe and effective and available in many countries, making them one of the swiftest-made vaccines for an emergent infection to date [13]. For many members of the public, the COVID-19 vaccine’s brief production timeline leaves space for doubt [13]. Another leading cause of hesitancy comes from the rapid increase of conspirative or influenced communication and information concerning the vaccine [14]. This generates an atmosphere that makes the community suspicious and skeptical of SARS-CoV-2 vaccination, including HCP [15,16,17,18,19]. There is a problem regarding the various scales and indices offered to quantify hesitancy in various target groups, for example, parents, healthcare personnel, chronic patients, or caregivers. The WHO SAGE Working Group on Vaccine Hesitancy created a ten-item Vaccine Hesitancy Scale (VHS) to be extensively used in diverse backgrounds and countries [20]. The VHS is an all-purpose sufficient scale to be useful in many contexts; it has been verified and psychometrically assessed using survey data from parents who were questioned about their children’s vaccines [21,22]. Investigations on adult vaccinations are typically carried out in high-income countries and use instruments with typical frameworks or using scoping reviews rather than scales [23,24,25]. There is a lack of data on adult vaccine hesitancy, without scales that are adjustable to these circumstances. Therefore, while the literature on SARS-CoV-2 vaccine hesitancy expands, more research on other vaccine-preventable diseases is crucial. Akel and colleagues carried out a modification of a VHS for use in adult vaccination in the United States and China [1] to evaluate the correlation between vaccine hesitancy and SARS-CoV-2. Furthermore, the adult Vaccine Hesitancy Scale (aVHS) is valid and reliable for evaluating attitudes toward other vaccine preventable diseases (VPDs). 

The 10-item aVHS has a 5-point Likert scale as answer options, ranging from least hesitant (1) to most hesitant (5).

The aim of the present study was to evaluate the cross-cultural adaptation, reliability, and validity of the Italian version of the aVHS.

## 2. Materials and Methods

### 2.1. Study Design

A prospective and cross-sectional study was performed in the context of periodic occupational health surveillance by University of Catania, Department of clinical and experimental medicine, using convenience and snowball sampling [26]. The inclusion criteria for recruiting workers were: (1) Italian literate individuals, (2) ≥18 year. The exclusion criteria were: (1) conditions that prevent work activity; (2) comorbidities that affect functional status; (3) cognitive disorders.

All participants were enrolled in October 2021 and completed for the first time the aVHS. In December 2021, the second administration of the aVHS questionnaire was completed to confirm reliability.

Socio-demographic data of workers were evaluated to reveal their interactions to the cultural adaptation process. 

The sample size of the study was determined according to Fayers and Machin suggestions [27]. The sample size should be at least five times the item number in cultural adaptation, validity, and reliability studies, and also at least more than 100 [27]. Accordingly, 160 workers were enrolled to evaluate the reliability and consistency of the questionnaire. The sample size for the reproducibility was calculated with the G* power 3 software with an effect size of 0.4, a probability of error = 0.05, and the power of 0.80 [28]. In detail, the workers were divided, arbitrarily, into four groups: (1) n.40 HCP; (2) n.40 Master’s degree graduates; (3) n.40 high-school graduation diploma; and 4) n.40 with only compulsory schooling. 

### 2.2. Cross-Cultural Adaptation

The cross-cultural adaption process was carried out according to guidelines proposed by Sousa and Rojjanasrirat [29]. At the same time, the translation of the original version of the aVHS, available online [30], into Italian was finalized, in agreement with the guidelines for translation of questionnaires [31]. The detailed stages are as follows. 

The English version of the aVHS was separately translated into Italian by two native Italians with a good knowledge of English. Neither of the translators had knowledge of the aVHS before. Next, the two translated aVHS (Italian versions) were matched, fused, and then assessed with the original version. After this, the combined Italian version was sent back to the translators to be translated into English, separately. A back-translation version (English) was obtained and after an expert committee (researchers and translators) examined the translations and resolved incongruities. 

A pilot study was carried out administering the questionnaire to 25 workers of various backgrounds to evaluate the pre-final version of the Italian aVHS. All workers were asked to report any unclear elements of the questionnaire. Subsequently the commission of experts met again to evaluate the final questionnaire further. The translated questionnaire is in the supplementary material of this research.

### 2.3. Reliability and Validity

The intra-class correlation coefficient (ICC) was evaluated to measure the sensitivity and reproducibility of the scale. An ICC value superior to 0.80 showed acceptable reproducibility [32]. 

The internal consistency (IC) was calculated using the Cronbach alpha coefficient test, and rates ranging from 0.7 to 0.95 indicate a good internal consistency. 

The scale content of validity index (S-CVI) was considered acceptable when the S-CVI was at least 0.90 [33].

**Figure 1:**
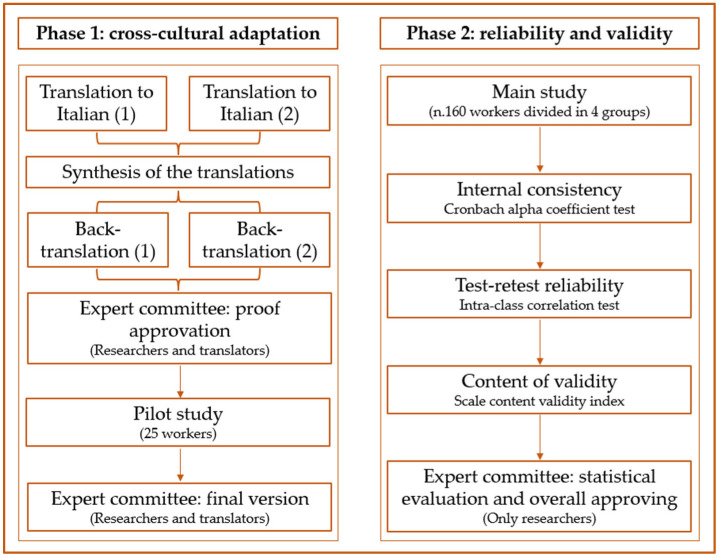
Cross-cultural adaptation, reliability, and validity of the Italian version of adult Vaccine Hesitancy Scale (aVHS) for the working-age population.

Figure 1 summarizes the cross-cultural adaptation, reliability, and validity of the Italian version of the aVHS process. 

### 2.4. Statistical Analysis

All statistical analyses were carried out using *jamovi* software (version 2.2.5 for Windows) [34]. 

Mean and standard deviation were presented for quantitative variables. Percentages were given for qualitative variables. 

## 3. Results

A total of 160 workers completed the aVHS questionnaire from October to December 2021. As specified in materials and methods, the workers were divided into four groups according to profession if HCP or the level of education for the other three groups. Characteristics are presented in Table 1.

The ANOVA aVHS score highlights a statistically significant difference among the four groups (R^2^ 0.4514; *p*-value < 0.0001).

Results of the ICC were analyzed on questionnaires administered twice at a distance of two months and revealed a satisfactory reproducibility (0.87). 

The IC of the aVHS was assessed by the Cronbach alpha coefficient test, that results 0.94 demonstrated an excellent IC reliability. The S-CVI calculated for the total scale was 0.97. Table 2 summarizes the results of internal consistency, reliability, and validity.

## 4. Discussion

This research, after evaluating the cross-cultural adaptation, explored the internal consistency, reliability, and validity of the Italian version of the aVHS. 

The scale demonstrates an excellent IC, making the scale reliable as a tool for evaluating vaccine hesitancy in adults. 

In addition, results highlight that the aVHS shows both concurrent and content. Although there is no standard method to quantify vaccine hesitancy, a VHS should be used dichotomously. Indeed, the frequency of vaccine acceptance, be it for influenza or COVID-19 or other VPDs, is significantly lower in vaccine-hesitant individuals. This model was examined using the dichotomous predictor [1]. 

Our study of the four groups of workers revealed that HCPs are less hesitant than other workers, obviously because they are more educated and have suffered the SARS-CoV-2 pandemic for the past two years.

Furthermore, the aVHS questionnaire should be used in several different settings, especially to prevent and promote health in the workplace. It is useful for detecting any reasons that distance adults from vaccination.

The aVHS has already been used in the survey from different countries. A study in India investigated COVID-19 and non-COVID-19 vaccine hesitancy, psychosocial aspects, measures, and individual-level vaccine hesitancy interventions among perinatal women [35]. Zhang et al. [36] carried out a study to assess if actions and vaccine decision-making could contribute to the worldwide spread of infectious diseases, through internet-based surveys from people living in the United States, China, Taiwan, Malaysia, Indonesia, and India. Moreover, the aVHS helped to assess the degree to which US parents are expected to get their children vaccinated against COVID-19 and recognize parental worries about vaccines [37]. Another investigation used the aVHS to identify the main barriers to vaccine acceptance among medical students in Kazakhstan [38]. The aVHS was used to assess vaccination hesitancy against herpes zoster, influenza, and pneumonia among adults [39,40].

The aVHS could be used together with other tests, for instance, psychometric evaluations, in this case it could highlight how some psychosocial characteristics can influence vaccine outcome. Investigation to date has shown that the reasons for and expressions of vaccine hesitancy are extremely different [20] and need to be better known in order to properly focus on emerging concerns. Justifications for hesitancy can differ depending on the particular vaccine or vaccines in subject, the persons or groups expressing unwillingness, and the context. Tools are necessary to assess the scope and scale of hesitancy issues by vaccine and background. Preferably, a common survey tool that can be used worldwide would allow comparability across territories.

Researching the global impact of vaccine hesitancy—including willingness to accept COVID-19 vaccines—could be complicated by the multifaceted nature of this phenomenon [3]. This involves the presence of cognitive, psychologic, socio-demographic and cultural aspects that impact to vaccine hesitancy [41,42,43,44]. Assessment of such factors is crucial to focus on COVID-19 vaccine hesitancy, after the evaluation of the scope and scale of this public health risk [45]. This can help in guiding interventional measures aimed at building and maintaining responses to tackle this threat [46].

There are some limitations in the present study. Firstly, a convenience sampling method was used, and the participants were recruited from only one occupational medicine unit. Moreover, we excluded adults with disorders, cognitive impairment, or inability which prohibit having an active working life. 

## 5. Conclusions

In conclusion, the aVHS is a valid and reliable tool for evaluating vaccine hesitancy toward adult vaccinations. We suggest the use of this scale in upcoming surveys that increase the opinions and perceptions of adult vaccinations. 

During such a crucial time as the current COVID-19 pandemic, this could represent a key instrument, since it is capable of evaluating how adult approaches change over time during the current COVID-19 vaccination campaigns. 

Finally, the aVHS could represent a valuable tool to know how these pandemic influences future vaccine decision-making and respond to such an issue.

## Figures and Tables

**Table 1:** Worker’s characteristics (n.160).

Variable	Group 1 * n.40	Group 2 * n.40	Group 3 * n.40	Group 4 * n.40
Gender				
F (n. %)	20 (50%)	19 (47%)	21 (53%)	21 (53%)
Age (mean ± SD)	44.3 ± 9.8	42.8 ± 8.3	45.7 ± 9.1	44.0 ± 8.6
Occupation				
Employee (n. %)	31 (77%)	25 (62%)	33 (82%)	36 (90%)
Chief/Head (n. %)	4 (10%)	9 (22%)	0 (0%)	0 (0%)
Entrepreneur (n. %)	1 (2%)	1 (3%)	2 (5%)	0 (0%)
Freelance (n. %)	3 (8%)	4 (10%)	4 (10%)	2 (5%)
Unemployed (n. %)	1 (3%)	1 (3%)	1 (3%)	2 (5%)
aVHS (mean ± SD)	41 ± 5 ^^^	29 ± 9 ^^^	26 ± 4 ^^^	31 ± 6 ^^^

* Groups: (1) n.40 HCP; (2) n.40 Master’s degree graduates; (3) n.40 high-school graduation diploma; and (4) n.40 with only compulsory schooling. ^^^ Statistically significant difference.

**Table 2:** Internal consistency, reliability, and validity of the Italian version of adult Vaccine Hesitancy Scale (aVHS) for the working-age population.

	ICC * (95% CI)	IC (α) °	S-CVI ^
Total Scale	0.87 (0.63–0.96)	0.94	0.97

* ICC = Intra-class correlation coefficient. ° Internal consistency (Cronbach alpha coefficient test). ^ scale content of validity index.

## Data Availability

The data presented in this study are available on request from the corresponding author.

## Supplementary Materials

The following are available online at https://www.mdpi.com/article/10.3390/vaccines10020224/s1, Table S1: Italian version of aVHS.

## References

[1] Akel K.B., Masters N.B., Shih S.-F., Lu Y., Wagner A.L. (2021). Modification of a Vaccine Hesitancy Scale for Use in Adult Vaccinations in the United States and China. Hum. Vaccines Immunother..

[2] Callender D. (2016). Vaccine Hesitancy: More than a Movement. Hum. Vaccines Immunother..

[3] MacDonald N.E. (2015). Vaccine Hesitancy: Definition, Scope and Determinants. Vaccine.

[4] Rapisarda V., Nunnari G., Senia P., Vella F., Vitale E., Murabito P., Salerno M., Ledda C. (2019). Hepatitis B Vaccination Coverage among Medical Residents from Catania University Hospital, Italy. Future Microbiol..

[5] Rapisarda V., Bracci M., Nunnari G., Ferrante M., Ledda C. (2014). Tetanus Immunity in Construction Workers in Italy. Occup. Med..

[6] Costantino C., Ledda C., Squeri R., Restivo V., Casuccio A., Rapisarda V., Graziano G., Alba D., Cimino L., Conforto A. (2020). Attitudes and Perception of Healthcare Workers Concerning Influenza Vaccination during the 2019/2020 Season: A Survey of Sicilian University Hospitals. Vaccines.

[7] Ledda C., Rapisarda V., Maltezou H.C., Contrino E., Conforto A., Maida C.M., Tramuto F., Vitale F., Costantino C. (2021). Coverage Rates against Vaccine-Preventable Diseases among Healthcare Workers in Sicily (Italy). Eur. J. Public Health.

[8] Ledda C., Cinà D., Garozzo S.F., Senia P., Consoli A., Marconi A., Scialfa V., Nunnari G., Rapisarda V. (2019). Tuberculosis Screening among Healthcare Workers in Sicily, Italy. Future Microbiol..

[9] Ledda C., Cinà D., Garozzo S.F., Vella F., Consoli A., Scialfa V., Proietti L., Nunnari G., Rapisarda V. (2019). Vaccine-Preventable Disease in Healthcare Workers in Sicily (Italy): Seroprevalence against Measles. Future Microbiol..

[10] Dubé E., Gagnon D., Nickels E., Jeram S., Schuster M. (2014). Mapping Vaccine Hesitancy—Country-Specific Characteristics of a Global Phenomenon. Vaccine.

[11] Opel D.J., Taylor J.A., Zhou C., Catz S., Myaing M., Mangione-Smith R. (2013). The Relationship Between Parent Attitudes About Childhood Vaccines Survey Scores and Future Child Immunization Status: A Validation Study. JAMA Pediatr..

[12] Ledda C., Costantino C., Motta G., Cunsolo R., Stracquadanio P., Liberti G., Maltezou H.C., Rapisarda V. (2022). SARS-CoV-2 MRNA Vaccine Breakthrough Infections in Fully Vaccinated Healthcare Personnel: A Systematic Review. TropicalMed.

[13] Dror A.A., Eisenbach N., Taiber S., Morozov N.G., Mizrachi M., Zigron A., Srouji S., Sela E. (2020). Vaccine Hesitancy: The next Challenge in the Fight against COVID-19. Eur. J. Epidemiol..

[14] Brennen J.S., Simon F., Howard P., Nielsen R.K. Types, Sources and Claims of COVID-19 Misinformation. https://reutersinstitute.politics.ox.ac.uk/types-sources-and-claims-covid-19-misinformation.

[15] Maltezou H.C., Pavli A., Dedoukou X., Georgakopoulou T., Raftopoulos V., Drositis I., Bolikas E., Ledda C., Adamis G., Spyrou A. (2021). Determinants of Intention to Get Vaccinated against COVID-19 among Healthcare Personnel in Hospitals in Greece. Infect. Dis. Health.

[16] Lu F., Sun Y. (2022). COVID-19 Vaccine Hesitancy: The Effects of Combining Direct and Indirect Online Opinion Cues on Psychological Reactance to Health Campaigns. Comput. Hum. Behav..

[17] Hawlader M.D.H., Rahman M.L., Nazir A., Ara T., Haque M.M.A., Saha S., Barsha S.Y., Hossian M., Matin K.F., Siddiquea S.R. (2022). COVID-19 Vaccine Acceptance in South Asia: A Multi-Country Study. Int. J. Infect. Dis..

[18] Walker K.K., Head K.J., Owens H., Zimet G.D. (2021). A Qualitative Study Exploring the Relationship between Mothers’ Vaccine Hesitancy and Health Beliefs with COVID-19 Vaccination Intention and Prevention during the Early Pandemic Months. Hum. Vaccines Immunother..

[19] Ledda C., Costantino C., Cuccia M., Maltezou H.C., Rapisarda V. (2021). Attitudes of Healthcare Personnel towards Vaccinations before and during the COVID-19 Pandemic. IJERPH.

[20] Larson H.J., Jarrett C., Schulz W.S., Chaudhuri M., Zhou Y., Dube E., Schuster M., MacDonald N.E., Wilson R. (2015). Measuring Vaccine Hesitancy: The Development of a Survey Tool. Vaccine.

[21] Domek G.J., O’Leary S.T., Bull S., Bronsert M., Contreras-Roldan I.L., Bolaños Ventura G.A., Kempe A., Asturias E.J. (2018). Measuring Vaccine Hesitancy: Field Testing the WHO SAGE Working Group on Vaccine Hesitancy Survey Tool in Guatemala. Vaccine.

[22] Shapiro G.K., Tatar O., Dube E., Amsel R., Knauper B., Naz A., Perez S., Rosberger Z. (2018). The Vaccine Hesitancy Scale: Psychometric Properties and Validation. Vaccine.

[23] Quinn S.C., Jamison A., Freimuth V.S., An J., Hancock G.R., Musa D. (2017). Exploring Racial Influences on Flu Vaccine Attitudes and Behavior: Results of a National Survey of White and African American Adults. Vaccine.

[24] Bricout H., Torcel-Pagnon L., Lecomte C., Almas M.F., Matthews I., Lu X., Wheelock A., Sevdalis N. (2019). Determinants of Shingles Vaccine Acceptance in the United Kingdom. PLoS ONE.

[25] Alabbad A.A., Alsaad A.K., Al Shaalan M.A., Alola S., Albanyan E.A. (2018). Prevalence of Influenza Vaccine Hesitancy at a Tertiary Care Hospital in Riyadh, Saudi Arabia. J. Infect. Public Health.

[26] Goodman L.A. (1961). Snowball Sampling. Ann. Math. Stat..

[27] Fayers P.M., Machin D. (2016). Quality of Life: The Assessment, Analysis and Reporting of Patient-Reported Outcomes.

[28] Faul F., Erdfelder E., Lang A.-G., Buchner A. (2007). G*Power 3: A Flexible Statistical Power Analysis Program for the Social, Behavioral, and Biomedical Sciences. Behav. Res. Methods.

[29] Sousa V.D., Rojjanasrirat W. (2011). Translation, Adaptation and Validation of Instruments or Scales for Use in Cross-Cultural Health Care Research: A Clear and User-Friendly Guideline: Validation of Instruments or Scales. J. Eval. Clin. Pract..

[30] Wagner A. Adult Vaccine Hesitancy Scale. https://figshare.com/articles/online_resource/adult_Vaccine_Hesitancy_Scale/13207145.

[31] Beaton D.E., Bombardier C., Guillemin F., Ferraz M.B. (2000). Guidelines for the Process of Cross-Cultural Adaptation of Self-Report Measures. Spine.

[32] Terwee C.B., Bot S.D.M., de Boer M.R., van der Windt D.A.W.M., Knol D.L., Dekker J., Bouter L.M., de Vet H.C.W. (2007). Quality Criteria Were Proposed for Measurement Properties of Health Status Questionnaires. J. Clin. Epidemiol..

[33] Polit D.F., Beck C.T., Owen S.V. (2007). Is the CVI an Acceptable Indicator of Content Validity? Appraisal and Recommendations. Res. Nurs. Health.

[34] The Jamovi Project Jamovi (Version 2.2.5). https://www.jamovi.org.

[35] Ransing R., Kukreti P., Raghuveer P., Puri M., Paranjape A.D., Patil S., Hegde P., Padma K., Kumar P., Kishore J. (2022). A Brief Psycho-Social Intervention for COVID-19 Vaccine Hesitancy among Perinatal Women in Low-and Middle-Income Countries: Need of the Hour. Asian J. Psychiatry.

[36] Zhang F., Shih S.-F., Harapan H., Rajamoorthy Y., Chang H.-Y., Singh A., Lu Y., Wagner A.L. (2021). Changes in COVID-19 Risk Perceptions: Methods of an Internet Survey Conducted in Six Countries. BMC Res. Notes.

[37] Szilagyi P.G., Shah M.D., Delgado J.R., Thomas K., Vizueta N., Cui Y., Vangala S., Shetgiri R., Kapteyn A. (2021). Parents’ Intentions and Perceptions About COVID-19 Vaccination for Their Children: Results From a National Survey. Pediatrics.

[38] Bolatov A.K., Seisembekov T.Z., Askarova A.Z., Pavalkis D. (2021). Barriers to COVID-19 Vaccination among Medical Students in Kazakhstan: Development, Validation, and Use of a New COVID-19 Vaccine Hesitancy Scale. Hum. Vaccines Immunother..

[39] Lu X., Lu J., Zhang F., Wagner A.L., Zhang L., Mei K., Guan B., Lu Y. (2021). Low Willingness to Vaccinate against Herpes Zoster in a Chinese Metropolis. Hum. Vaccines Immunother..

[40] Lu X., Lu J., Zhang L., Mei K., Guan B., Lu Y. (2021). Gap between Willingness and Behavior in the Vaccination against Influenza, Pneumonia, and Herpes Zoster among Chinese Aged 50–69 Years. Expert Rev. Vaccines.

[41] Murphy J., Vallières F., Bentall R.P., Shevlin M., McBride O., Hartman T.K., McKay R., Bennett K., Mason L., Gibson-Miller J. (2021). Psychological Characteristics Associated with COVID-19 Vaccine Hesitancy and Resistance in Ireland and the United Kingdom. Nat. Commun..

[42] Pomares T.D., Buttenheim A.M., Amin A.B., Joyce C.M., Porter R.M., Bednarczyk R.A., Omer S.B. (2020). Association of Cognitive Biases with Human Papillomavirus Vaccine Hesitancy: A Cross-Sectional Study. Hum. Vaccines Immunother..

[43] Browne M., Thomson P., Rockloff M.J., Pennycook G. (2015). Going against the Herd: Psychological and Cultural Factors Underlying the ‘Vaccination Confidence Gap’. PLoS ONE.

[44] Hornsey M.J., Harris E.A., Fielding K.S. (2018). The Psychological Roots of Anti-Vaccination Attitudes: A 24-Nation Investigation. Health Psychol..

[45] Lin C., Tu P., Beitsch L.M. (2020). Confidence and Receptivity for COVID-19 Vaccines: A Rapid Systematic Review. Vaccines.

[46] De Figueiredo A., Simas C., Karafillakis E., Paterson P., Larson H.J. (2020). Mapping Global Trends in Vaccine Confidence and Investigating Barriers to Vaccine Uptake: A Large-Scale Retrospective Temporal Modelling Study. Lancet.

