# Supplementary material for: The Italian Version of the Adult Vaccine Hesitancy Scale (aVHS) for the Working-Age Population: Cross-Cultural Adaptation, Reliability, and Validity
Source: Vaccines (Basel). 2022 Jan 31;10(2):224. doi: 10.3390/vaccines10020224 (PMC8874655; doi:10.3390/vaccines10020224)
Supplement: Supplementary file 1 [file vaccines-10-00224-s001.zip › vaccines-1553432-supplementary.pdf]

Supplementary Materials

Table S1. Italian version of aVHS.

L1	I vaccini sono importanti per la mia salute
L2	I vaccini sono efficaci
L3	Essere vaccinati è importante per la salute degli altri della mia comunità
L4	Tutte le vaccinazioni di routine raccomandate dal Ministero sono utili
L5	I nuovi vaccini comportano più rischi dei vecchi vaccini
L6	Le informazioni che ricevo sui vaccini dal CDC sono affidabili e degne di fiducia
L7	Accedere ai vaccini è un buon modo per proteggermi dalle malattie
L8	Generalmente, eseguo le indicazioni del mio medico curante a riguardo ai vaccini
L9	Sono preoccupato per i gravi effetti avversi dei vaccini
L10	Non ho bisogno di vaccini per malattie che non sono più comuni
